# Single-Cell Profiling to Explore Immunological Heterogeneity of Tumor Microenvironment in Breast Cancer

**DOI:** 10.3389/fimmu.2021.643692

**Published:** 2021-02-25

**Authors:** Xiao Yuan, Jinxi Wang, Yixuan Huang, Dangang Shangguan, Peng Zhang

**Affiliations:** ^1^Changsha KingMed Center for Clinical Laboratory Co., Ltd, Changsha, China; ^2^First Affiliated Hospital of Hunan University of Traditional Chinese Medicine, Changsha, China; ^3^Division of Immunotherapy, Institute of Human Virology, University of Maryland School of Medicine, Baltimore, MD, United States; ^4^Hunan Cancer Hospital, Changsha, China

**Keywords:** single-cell sequencing, breast cancer, single cell mass cytometry, tumor microenviroment, immune cell

## Abstract

Immune infiltrates in the tumor microenvironment (TME) of breast cancer (BRCA) have been shown to play a critical role in tumorigenesis, progression, invasion, and therapy resistance, and thereby will affect the clinical outcomes of BRCA patients. However, a wide range of intratumoral heterogeneity shaped by the tumor cells and immune cells in the surrounding microenvironment is a major obstacle in understanding and treating BRCA. Recent progress in single-cell technologies such as single-cell RNA sequencing (scRNA-seq), mass cytometry, and digital spatial profiling has enabled the detailed characterization of intratumoral immune cells and vastly improved our understanding of less-defined cell subsets in the tumor immune environment. By measuring transcriptomes or proteomics at the single-cell level, it provides an unprecedented view of the cellular architecture consist of phenotypical and functional diversities of tumor-infiltrating immune cells. In this review, we focus on landmark studies of single-cell profiling of immunological heterogeneity in the TME, and discuss its clinical applications, translational outlook, and limitations in breast cancer studies.

## Introduction

Born in 2009 (1), selected as the Method of the Year 2013 by Nature Methods (2), the single-cell sequencing technologies are revolutionizing the details of whole-transcriptome and proteome snapshots from a tissue to a cell (3–5). Compared with traditional bulk sequencing approaches, the single-cell sequencing technologies enable the identification of cellular heterogeneity in greater detail than conventional methods at the single-cell level. It shows unequaled strength in exploring cellular diversity especially immunological heterogeneity in the TME, which is an extremely subtle system and contains a variety of tumor cells and infiltrating immune cells (6, 7). Recently rapid developed single-cell RNA sequencing (scRNA-seq) methods have allowed for the identification of rare and novel cell types, simultaneous characterization of multiple different cell states, more accurate and integrated understanding of their roles in the tumor microenvironment. The workflow of scRNA-seq consists of single-cell capture, mRNA reverse transcription, cDNA amplification, library preparation, high-throughput sequencing, and data analysis. The number of sequenced reads, which represents the gene expression level, has been counted as a digital gene expression matrix for bioinformatic analysis (8, 9). In this review, we will outline the recent findings on tumor-infiltrating immune cells based on scRNA-seq in human breast cancers, and their connections with immunotherapy and potential clinical applications. We also explore ways in which other single-cell approaches, such as single-cell mass cytometry (10), that deepen our understanding of immunological responses and resistance in the tumor microenvironment, and examine potential future innovations in the field.

## Decomposition of Tumor Immune Microenvironment Using scRNA-seq

**Figure 1:**
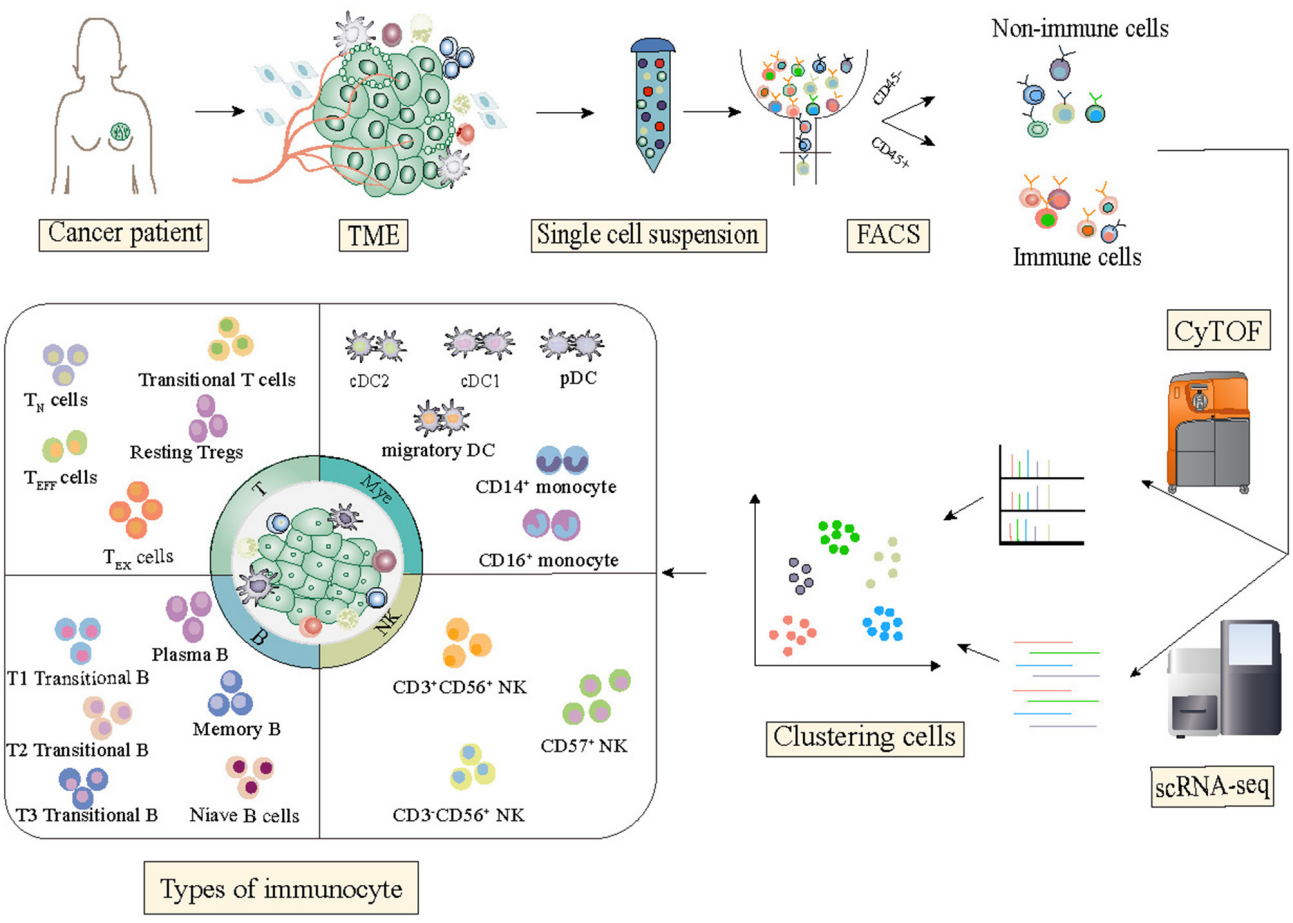
State of the art of single-cell technology and its application in breast cancer studies. Single-cell sequencing technologies have been designed for almost all the molecular layers of genetic information flow from RNA to proteins. For each molecular layer, multiple technologies have been developed, all of which have specific advantages and disadvantages. Single-cell technologies are close to comprehensively depicting the state of the functional properties and dynamic changes of immune cells in the tumor microenvironment.

Although the tumor–immune ecosystem is highly complex and comprises a heterogeneous collection of cells, single-cell RNA sequencing technology has emerged as a powerful tool for the dissection of the tumor immune microenvironment that uncovers the mechanism of activation, regulation, and communication (Figure 1).

## The Complexity of Tumor-Infiltrating Lymphocytes (TILs)

A research group from Australia analyzed intratumoral T cells isolated from tumor tissues by using the multiparameter flow cytometry method, based on a prospective cohort of 123 breast cancer patients, and showed that significant heterogeneity existed in the infiltrating T lymphocytes populations (11). Then, they performed single-cell transcriptome analysis on 6,311 flow-sorted CD3^+^CD45^+^ T cells from two samples of human primary triple-negative breast cancer (TNBC) tumor. A total of 10 distinct cell clusters included 3 CD8^+^ T cell clusters and 4 CD4^+^ T cell clusters were identified. Of interest, among the CD8^+^ T cell clusters, one cluster had the expression of molecules suggestive of a tissue-resident memory T (T_RM_) cell phenotype. This CD8^+^CD103^+^ T_RM_-like cluster was highly distinct, with 400 genes including several hallmarks of T_RM_ differentiation that statistically differentially expressed when compared with the other T cell clusters, and highly expressed both immune checkpoint molecules (such as *PDCD1* and *CTLA4*) and cytotoxic effector proteins (such as *GZMB* and *PRF1*). Moreover, the gene signatures of the CD8^+^ T_RM_ cluster that confirmed by using bulk RNA-seq data, were found to significantly correlate with favorable patient survival in early-stage TNBC. As indicated in this study, scRNA-seq enabled the discovery of minor subgroups of TILs that were related to immune-suppression or immune-surveillance, and biomarkers of these distinct immune cells may serve as prognostic factors or therapeutic targets for breast cancer. The main limitation of this study is that there were only two TNBC tumor samples profiled by scRNAseq.

It is clear that T cells have a dominant role in the tumor immune microenvironment, however, there is a growing appreciation of other components of TILs such as B cells may also contribute to anti-tumor immunity. Recently, Lu et al. (12) observed a phenotype switch of B cells during neoadjuvant chemotherapy that could enhance tumor-specific T cell responses. scRNA-seq of tumor-infiltrating B cells was performed in paired clinical samples of pre- (998 cells) and post-neoadjuvant chemotherapy (1,499 cells) collected from 4 breast cancer patients. The analytic result showed that a distinct B cell subset that expressed high levels of inducible T-cell co-stimulator ligand (*ICOSL*) significantly increased after neoadjuvant chemotherapy. Besides, the high expression of *CR2* and low expression of *IL-10* were also found in this special cell subset. The comparison between patients with stable diseases or progression and patients with partial or complete remission indicated that this cell subset was related to improved therapeutic efficacy. Further survival analyses indicated that ICOSL^+^ B cell abundance was an independent positive prognostic factor. They also identified the *CD55*, expressed by tumor cells, as the key factor determining the subset switch and conflicting roles of tumor-infiltrating B cells during chemotherapy. It was proposed that this chemotherapy-associated subset of B cells could promote tumor-specific T cell proliferation and reduce regulatory T cells (Tregs). Collectively, this study uncovered a new role of complement in B-cell-dependent anti-tumor immunity and indicated that *CD55* induced chemo-resistance by impeding the induction of ICOSL^+^ B cells and thus could be a potential therapeutic target to enhance the efficacy of immunogenic chemotherapy. However, their sub-stratified analysis and clinical conclusions should be validated in the future hypothesis-testing experimental investigation because of the small sample size examined in this study.

## Characterizing Immune Cell Heterogeneity

One of the most early-stage scRNA-seq studies for comprehensive profiling of breast cancer microenvironment was conducted by the Samsung Genome Institute (13). Researchers analyzed 515 cells from 11 patients representing the four subtypes of breast cancer: luminal A; luminal B; HER2; and triple-negative breast cancer (TNBC). The results revealed that after the separation of carcinoma cells via RNA-seq-inferred tumor-specific copy number variations, most of the non-cancer cells are immune cells because of their high scoring of the immune signatures. 175 tumor-associated immune cells were identified and further annotated as 3 distinct clusters including T lymphocytes, B lymphocytes, and macrophages by using immune cell type-specific gene sets. Interestingly, T cells and macrophages both display immunosuppressive characteristics: T cells with a regulatory or an exhausted phenotype and macrophages with an M2 phenotype. These immune cells with the expression of many immunosuppressive genes could promote tumorigenesis and restrain immune surveillance. Although the number of profiled cells was low and the sequencing depth was limited, this work demonstrated the feasibility of a comprehensive characterization of the heterogeneous immunological microenvironment of breast cancer samples by large-scale single-cell gene expression profiling protocol. Recently, Bao et al. (14) also described the molecular characteristics of M2-like TAM in the TME of breast cancer and identified the association of the immune landscape with clinical outcomes in TNBC by using an integrative analysis approach of combined single-cell and bulk tissue transcriptome profiling.

Another sophisticated TME profiling work provided by a team from the Memorial Sloan Kettering Cancer Center drew a single-cell atlas of diverse immune phenotypes of breast cancer samples and found the immune phenotype was associated with the tissue of residence (15). By assessing 45,000 cells captured from breast carcinomas, as well as matched normal breast tissue, blood, and lymph nodes of 8 treatment-naive patients, they identified 38 T cell, 27 myeloid lineage, 9 B cell, and 9 NK cell clusters, and observed several phenomena via data analysis: (1) T cells in blood and lymph node exhibited dissimilar phenotypes compared with T cells in breast tissue; (2) T and myeloid lineage cells exhibited considerable phenotypic overlap between tumor and normal tissue samples, but increased phenotypic heterogeneity and expansion of cell populations in the tumor was also observed; (3) Naive T cells were strongly enriched in 3 blood-specific clusters, while B cells were more prevalent in the lymph node than in other tissues; (4) A subset of T cell clusters was present in both tumor and normal tissue, but cytotoxic T cell clusters were more abundant in the tumor, as were Treg clusters; (5) Some myeloid clusters were shared between normal and tumor tissue, whereas clusters of more activated macrophages were specific to the tumor. Their results support a model of continuous activation and expansion (shaped by TCR specificity) in T cells and do not comport with the macrophage polarization model in the tumor microenvironment. Moreover, these findings offered a more nuanced view into the association between immune phenotypes and the tissues of residence and suggested that the immunological landscape based on the blood or normal samples may not reflect the functional and phenotypic diversity in the TME.

## Spatial Mapping of Single-Cell RNA-seq Data

While scRNA-seq has mainly been used to delineate cell subpopulations and their lineage relationships, recently developed spatial transcriptomics technologies have been designed to infer cell-cell communications and spatial architecture in the tumor microenvironment. A research group from Sweden employed an in-house spatial transcriptomics method to resolve spatial immune cell distribution from tumor tissue sections of BRCA patients diagnosed with HER2^+^ subtype (16, 17). The abundance and distribution of the infiltrated immune cells in different regions of the tumor tissue including invasive cancer regions were determined. Then the researchers combined the cross-sectioning and computational alignment to build three-dimensional images of the transcriptional map of the tumor microenvironment. This spatial transcriptomic landscape demonstrated the heterogeneous nature of tumor-immune interactions and reveal interpatient differences in TME patterns of breast cancer. To our knowledge, this is the first attempt to present a spatial map of comprehensive transcriptomics data from human breast cancer tissues and gain new insight into the immunological heterogeneity.

## Dissecting the Tumor Microenvironment Using Single-Cell Mass Cytometry

Single-cell RNA-seq captures the expression of thousands of genes, but at the cost of sparse data. In comparison, although mass cytometry measures a limited number of pre-selected markers, these markers are backed with decades of experimental experience, which makes mass cytometry an effective and efficient way to define cellular heterogeneity and a key complement to scRNA-seq (2, 18, 19).

To investigate immunological features of TME and their associations with clinical characteristics of breast cancer, Wagner et al. (20) provided a large-scale single-cell atlas of the human breast cancer tumor microenvironment by analyzing 144 human BRCA tumors covering all clinical subtypes and 50 non-tumor tissue samples by using single-cell mass cytometry. Through tumor and immune cell-centric antibody panels, a total of 73 proteins in 26 million cells was evaluated. Researchers observed significant differences in the T cell landscape of ER^−^ and ER^+^ tumors. In more than half of ER^−^ tumors but only 12% of ER^+^ tumors, over 10% of T cells expressed PD-1. For cell level, distinct PD-1^+^ phenotypes were separately enriched: PD-1^high^CTLA-4^+^CD38^+^ T cells were more frequent in ER^−^ tumors, whereas PD-1^int^CTLA-4^−^CD38^−^ T cells were enriched in ER^+^ tumors. This observation support that patients with ER- tumors are more suitable candidates for immunotherapy (21). They also observed high frequencies of PD-L1^+^ tumor-associated macrophages and exhausted T cells were found in high-grade ER^+^ and ER^−^ tumors, suggesting a possible association between an immunosuppressed environment and poor-prognosis of high-grade tumors. This sophisticated work enhanced our comprehension of the immune ecosystem of human breast cancer and revealed that TME-based stratification will facilitate the identification of BRCA patients for precision medicine approaches targeting the tumor and its immune environment. However, there are still some limitations in this study, and the dominant one is a lack of correlation analysis between their ecosystem-based patient grouping and clinical outcome or treatment response of BRCA patients.

Another impressive research work performed by Jackson et.al depicted the first single-cell pathology landscape of breast cancer by using the imaging mass cytometry (IMC) technology (22, 23). By the use of a designed breast tissue-specific IMC histology panel, a total of 855,668 cells in 381 images (289 tumors, 87 healthy breasts, and 5 liver controls) were been investigated with 35 antibodies simultaneously quantified. Cellprofiler (24) was used for single-cell feature extraction to obtain the expression level of marker genes. And PhenoGraph (25) was employed to identify the 27 meta clusters which represented various immune, stromal, and epithelial cell types. “Community” which consists of interactions between one or more cell phenotypes, was introduced to describe the complex multicellular interaction pattern. The Louvain community detection algorithm (26) was applied to identify highly interconnected spatial subunits in the tissue graph. Researchers investigated how the organization of single cells into communities contributes to the tissue architecture of breast cancer and its subtypes, and found cells from multiple meta clusters appeared in each clinically defined breast cancer subtype, which indicated the general classification based on pathology had limitations in explicate inter-and intrapatient cellular heterogeneity. Then they re-grouped patients based on their tumor cell meta cluster composition and identified 18 novel single-cell pathologies (SCP) subgroups using unsupervised clustering. This higher-resolution classification was then proved to be associated with distinct clinical outcomes. This study revealed that complex single-cell phenotypes and their spatial context could be reflected in the histological stratification and provided a basis for future study on spatial and phenotypic tissue features' influence on disease outcome. But, currently, the high complexity of data analysis for imaging mass cytometry approaches presents a major obstacle to the broad use of these methods in the scientific basic research and potential clinical use.

## Perspectives of Single-Cell Technologies in Breast Cancer Research

Although the heterogeneous cell populations in the TME stand out as the key barrier to delineate the tumor ecosystems, the advances in single-cell technologies, in particular scRNA-seq and mass cytometry, has revolutionized breast cancer research. The pioneering studies summarized in Table 1 have covered the development and applications of single-cell RNA sequencing and mass cytometry to address a wide range of topics such as intra-tumor heterogeneity of tumor samples, the characteristics of tumor microenvironments, and the mechanism of immunotherapy resistance. Improvement of existing single-cell sequencing technologies and the integration of single-cell sequencing with other high throughput and experimental protocols have provided powerful toolsets to understand many of the remaining mysteries of breast cancers.

**Table 1:** Summary table for the hallmark breast cancer studies using single-cell technologies.

	**Technology**	**Sample/data**	**Main findings**	**Clinical significance**	**References**
The complexity of tumor-infiltrating lymphocytes (TILs)	scRNA-seq (10X Genomics)	6,311 flow-sorted CD3+CD45+ T cells from two samples of TNBC	Discovery of minor subgroups of TILs that were related to immune-suppression	Biomarkers of the minor distinct TILs may serve as prognostic factors or therapeutic targets	(11)
	scRNA-seq (10X Genomics)	Paired samples of pre- (998 cells) and post-neoadjuvant chemotherapy (1,499 cells) collected from 4 BRCA patients	ICOSL^+^ B cells boost anti-tumor immunity by enhancing the effector to regulatory T cell ratio	The critical role of the B cell subset switch in chemotherapy response, which has implications in designing novel anti-cancer therapies.	(12)
Decomposition of tumor immune microenvironment using scRNA-seq	scRNA-seq (Fluidigm C1)	515 cells from 11 patients representing the four subtypes of breast cancer	T lymphocytes and macrophages both display immunosuppressive characteristics	The characteristics of different BRCA subtypes that are shaped by tumor cells and immune cells in TME	(13)
	scRNA-seq (inDrop); single-cell VDJ sequencing (10X Genomics)	45,000 cells captured in the normal and malignant breast tissues, lymph nodes, and peripheral blood of 8 treatment-naive patients	Despite the significant similarity between normal and tumor tissue-resident immune cells, continuous phenotypic expansions specific to the TME was observed	Support a model of continuous activation in T cells and do not comport with the macrophage polarization model in cancer	(15)
Spatial mapping of single-cell RNA-seq data	Spatial Transcriptomics (in-house)	Tumor tissue sections from BRCA patients diagnosed with HER2+ subtype	Demonstration of the heterogeneous nature of tumor-immune interactions and reveal interpatient differences in immune cell infiltration patterns	Potential for an improved stratification and description of the tumor-immune interplay, which is likely to be essential in treatment decisions	(16, 17)
Dissecting the tumor microenvironment using single-cell mass cytometry	Single-Cell Mass Cytometry	26 million cells from 144 human breast tumors including and 50 non-tumor tissue samples	Relationship analyses between tumor and immune cells revealed characteristics of TME related to immunosuppression and poor prognosis	TME-based classification of BRCA will facilitate the identification of individuals for precision medicine approaches	(20)
	Imaging mass cytometry	855,668 cells in 381 images (289 tumors, 87 healthy breasts, and 5 liver controls)	Multicellular features of TME and novel subgroups of breast cancer that are associated with distinct clinical outcomes	Spatially resolved, single-cell analysis can characterize intratumor phenotypic heterogeneity with the potential to inform patient-specific diagnosis	(23)

The advent of rapidly developing single-cell sequencing technologies are revolutionizing our ability to study tumor immunology, and these initial studies provided a proof of concept for the utility of single-cell profiling of TME. However, substantial limitations and challenges still exist in this approach. First, most single-cell technologies (such as single-cell RNA-sequencing) are very sensitive to the quality of sample collection and library construction, and therefore couldn't be applied to the profiling of sub-optimally preserved or handled clinical specimens (27, 28). Second, given the technological and throughput constraints of cellular captures, single-cell technologies usually profile only a partial sampling of tumor tissues. To what extent the sequenced cells represent the distribution of cells in the entire microenvironment is not clear. Third, high cost limits the ability to profile large cohorts of tumor samples, so most single-cell studies to date include a few patients, which limits the opportunity to investigate effects on clinical characteristics and outcomes. Spatial single-cell sequencing, single-cell proteomics, and single-cell epigenomics technologies are some of the major directions of single-cell sequencing technologies that will bring the second wave of revolutions of cancer research (29–31). Understanding the orchestrated organizations and interactions of cancer and immune cells in a spatial coordinate system will provide further insights into cancer progression and could provide clues for improving the efficiency of current immunotherapies.

Besides, the use of single-cell technologies in profiling the tumor microenvironment of breast cancers has been largely limited to basic research. Its potential for clinical utility including disease diagnosis, dynamical monitoring, therapeutic efficacy, and prognostic prediction is yet to be realized (32–34). First, the standardized procedures of sample processing for clinical use of single-cell sequencing technologies are urgently required. It is important to set measurable criteria and establish practical protocols for the processing of tissue sampling from operations (such as resection, selection, and isolation from tissue to single cells). Second, the methodologies and procedures of single-cell sequencing data pre-processing, quality control, data analysis, and visualizations of results need to be simplified. Besides, the most important issue for potential clinical use is how to interpret analysis results to clinicians and patients, and provide valuable information for clinical decision-making. We believe, in the near future, the promising clinical use based on developed single-cell technologies will improve the understanding of molecular pathogenesis and pathophysiology, and facilitate the discovery and validation of biomarkers and targets for breast cancer.

## Author Contributions

XY and JW contributed equally to researching, writing of initial drafts, and assembling manuscripts with the help of YH. DS and PZ conceptualized, edited, and assembled the final submitted manuscript. All authors contributed to the article and approved the submitted version.

## Conflict of Interest

XY was employed by the Changsha KingMed Center For Clinical Laboratory Co., Ltd. The remaining authors declare that the research was conducted in the absence of any commercial or financial relationships that could be construed as a potential conflict of interest.
